# Efficacy of doxorubicin-transferrin conjugate in apoptosis induction in human leukemia cells through reactive oxygen species generation

**DOI:** 10.1007/s13402-015-0256-2

**Published:** 2015-11-26

**Authors:** Marzena Szwed, Audrey Laroche-Clary, Jacques Robert, Zofia Jozwiak

**Affiliations:** 1 Department of Thermobiology, Faculty of Biology and Environmental Protection, University of Lodz, Pomorska 141/143 Street, 90-236 Lodz, Poland; 2 INSERM U916, Institut Bergonié, Université de Bordeaux, 33076 Bordeaux, France

**Keywords:** Doxorubicin-transferrin conjugate, Anticancer therapy, ROS generation, Leukemia cells, Mitochondrial membrane potential

## Abstract

**Background:**

Doxorubicin (DOX) is a small molecular cytotoxic agent that can be transferred efficiently to cancer cells by nanocarriers. This anthracycline antibiotic serves as an effective anti-neoplastic drug against both hematological and solid malignancies. Here, we set out to assess the capacity of a novel doxorubicin - transferrin conjugate (DOX-TRF) to provoke apoptosis in human normal and leukemia cells through free radicals produced via a redox cycle of doxorubicin (DOX) when released from its conjugate.

**Methods:**

After DOX-TRF exposure, we determined the time-course of apoptotic and necrotic events, the generation of reactive oxygen species (ROS), changes in mitochondrial membrane potential, as well as alterations in cytochrome *c* levels and intracellular calcium concentrations in human leukemia-derived cell lines (CCRF-CEM, K562 and its doxorubicin-resistant derivative K562/DOX) and normal peripheral blood-derived mononuclear cells (PBMC).

**Results:**

We found that DOX-TRF can induce apoptosis in all leukemia-derived cell lines tested, which was associated with morphological changes and decreases in mitochondrial membrane potential. In comparison to free DOX treated cells, we observed a time-dependency between a higher level of ROS generation and a higher drop in mitochondrial membrane potential, particularly in the doxorubicin-resistant cell line. In addition, we found that the apoptotic cell death induced by DOX-TRF was directly associated with a release of cytochrome *c* from the mitochondria and an increase in intracellular calcium level in all human leukemia-derived cell lines tested.

**Conclusions:**

Our data indicate that DOX-TRF is considerably more cytotoxic to human leukemia cells than free DOX. In addition, we show that DOX-TRF can effectively produce free radicals, which are directly involved in apoptosis induction.

## Introduction

During the last 15 years, numerous peptides and proteins have been used for improving the transport of cytotoxic agents. Chemical modifications of conventional chemotherapeutics provide novel possibilities for enhancing their limited clinical success so far, which may be due to tumor heterogeneity and the emergence of drug resistance. Several approaches have been tested, including drug entrapment in carriers like liposomes [1], polymeric micelles [2] and nanoparticles [3; 4]. More recent efforts have focused on the identification of new vehicles for intracellular delivery, such as lactosaminated human albumin [3] and transferrin [4].

A small molecular cytotoxic agent that can be transferred efficiently to cancer cells by nanocarriers is doxorubicin (DOX). This anthracycline antibiotic is an effective anti-neoplastic drug against both hematological malignancies and solid tumors [5]. The mechanism of DOX action has been linked to DNA damage, topoisomerase inhibition and iron sequestration with subsequent free radical generation [6–8]. However, its nonspecific distribution, leading to intolerable adverse effects and the development of drug resistance, still limits the current use of DOX.

Polymer-based delivery systems that have been developed for DOX are mostly designed to direct DOX away from sites of drug toxicity, especially the heart, and towards the site of drug action, i.e., the tumor [9, 10]. These delivery systems have mostly resulted in a modest increase in the therapeutic efficacy of DOX, usually in DOX sensitive cancers, in preclinical and clinical studies. With the aim to change the total DOX dose and to limit its high peak serum concentration, we synthesized a doxorubicin-transferrin (DOX-TRF) conjugate, a known approach to improve the efficiency and delivery of anthracycline antibiotics [11, 12]. Transferrin (TRF) possesses several benefits over other drug carriers, which makes this protein an ideal candidate for improving the anti-tumor properties of DOX. Firstly, it is not immunogenic, secondly, it is involved in iron uptake and the regulation of cellular growth and, thirdly, the expression of its receptors is significantly upregulated in a variety of malignancies. As a result, the conjugation of anticancer drugs with TRF may result in the delivery of cytotoxic agents directly to neoplastic cells, with a limited damage to normal cells [13].

Recently, we reported that DOX-TRF can overcome resistance of cancer cells to conventional chemotherapy regimens involving anthracycline drugs [14]. As yet, however, little is known about its potency to induce programmed cell death (apoptosis) in different types of cancer cells. Here, we addressed the question whether free radicals can participate in apoptosis induction by DOX-TRF using spectrometric, spectrofluorimetric, immunoenzymatic and microscopic methods. Specifically, we monitored the time-course of apoptotic and necrotic events, i.e., the production of reactive oxygen species (ROS), changes in mitochondrial membrane potential (Δψ_m_), changes in the level of intracellular calcium, release of cytochrome *c* to the cytosol, as well as morphological changes in both leukemia and normal cells in the presence and absence of an antioxidant, N-acetylcysteine (NAC). We show that DOX-TRF is more cytotoxic towards leukemia cells than normal blood cells. Our results indicate that the induction of apoptosis by DOX-TRF in human leukemia cells is related to the generation of free radicals and a perturbation of their redox homeostasis.

## Materials and methods

### Reagents and chemicals

RPMI-1640 culture medium, fetal bovine serum (FBS), penicillin-streptomycin antibiotics, L-glutamine and phosphate-buffered saline (PBS) were purchased from Lonza (Lievres, Belgium), whereas doxorubicin (DOX) was purchased from Sequoia Research Products (Pangbourne, United Kingdom). The XTT assay kit, H_2_DCF-DA, JC-1, and all reagents for carrying out the conjugation procedure were purchased from Sigma-Aldrich chemicals (Darmstadt, Germany). DOX was coupled to TRF using a modified conjugation procedure developed by Berczi et al. [15] and the conjugate obtained was analyzed by mass spectrometry [16]. Cytochrome *c*, YO-PRO-1 and Fluo-4 AM assay kits were obtained from Invitrogen (United Kingdom). The other chemicals were purchased from POCH S.A. (Gliwice, Poland) if not otherwise indicated. The tissue culture dishes were purchased from Corning (New York, USA).

### Cell lines and primary blood cells

Three human leukemia-derived cell lines were used for the in vitro studies: the acute lymphoblastic leukemia-derived cell line CCRF-CEM, the chronic erythroleukemia-derived cell line K562 and its doxorubicin-resistant derivative cell line K562/DOX [14]. In all experiments normal peripheral blood mononuclear cells (PBMC) were included. These latter cells were isolated from blood obtained from healthy young (23–35 years) non-smoking men by centrifugation in a Histopaque density gradient (300 *g* for 30 min at 22 °C). Both normal and leukemic cells were cultured in RPMI-1640 medium supplemented with L-glutamine (4 mM), penicillin (100 U/ml), streptomycin (100 μg/ml) and 10 % v/v FBS using standard conditions, i.e., at 37 °C in a humidified atmosphere containing 5 % CO_2_. In all experiments, cells in a logarithmic growth phase were used. The K562/DOX cell line was grown in the presence of 0.02 μM DOX as a selection agent. All cell lines were monitored periodically for mycoplasma contamination. In some of the experiments, cells were pre-incubated with the antioxidant N-acetylcysteine (NAC), 3 mM for 1 h, after which DOX or DOX-TRF at the appropriate concentrations were added and the incubation was continued for the required period of time under the same conditions. In the control experiments, cells were treated with a corresponding volume of PBS (instead of drugs or antioxidants) according to the same schedule.

### Quantification of viable cells by XTT assay

The principle of the XTT assay is that viable cells reduce the tetrazolium salt XTT (2,3-bis(2-methyloxy-4-nitro-5-sulfophenyl)-2H-tetrazolium-5 carboxanilide (Sigma-Aldrich) to an orange-colored water-soluble product [17]. Here, 10^4^ CCRF-CEM, K562 and K562/DOX cells or 10^5^ PBMC cells were seeded in each well of a 96-well microplate in 0.1 ml culture medium. Next, 0.05 ml DOX or DOX-TRF at different concentrations were added to the appropriate wells, and the cells were incubated with these drugs for 72 h. At the end of this incubation period, the cells were centrifuged (230 *g* for 10 min at 4 °C) and the medium was gently removed. Subsequently, 0.05 ml XTT at a final concentration of 0.3 mg/ml in medium was added to each well and the microplates were incubated for another 4 h. The resulting reduction of XTT was measured at 492 nm using a microtitre plate reader (Awareness Technology Inc., USA). The percentage of viable cells was calculated by comparing the reduction of XTT in drug treated cells to that in the untreated control cells. The cytotoxicity was expressed as IC_50_, which is the concentration at which the agent reduces the cell viability by 50 % relative to the control cells. These values were calculated using GraphPad Prism 4.03 software (GraphPad Inc.).

### ROS formation assay

To measure intracellular reactive oxygen species (ROS) formation, the fluorescent probe dichlorodihydrofluorescein diacetate (H_2_DCF-DA) was used. Briefly, PBMC, CCRF-CEM, K562 and K562/DOX cells were seeded in 96-well plates and incubated with drugs (DOX or DOX-TRF) for 3, 6, 12, 24, 48 and 72 h. Next, the cells were incubated with 5 μM H_2_DCF-DA at 37 °C for 30 min [18] and ROS fluorescence (DCF) was measured using a Fluoroskan Ascent FL microplate reader (Labsystems, Sweden).

### YO-PRO-1 iodide/propidium iodide apoptosis assay

During apoptosis, cell membranes become permeable to the green fluorescence dye YO-PRO-1 iodide, whereas they are not permeable to the red fluorescent dye propidium iodide (PI). After DOX or DOX-TRF treatment, a mixture of YO-PRO-1 iodide and PI (0.1 μM each) was added to the cells and the samples were incubated for 20 min on ice. Next, the samples were analyzed by flow cytometry (LSR II, Becton Dickinson) with an excitation at 488 nm to visualize the YO-PRO-1 green fluorescence and PI red fluorescence. Whereas after this treatment living cells exhibit a low level of green fluorescence, apoptotic cells gradually exhibit higher levels of green fluorescence. Dead cells exhibit both green and red fluorescence. The negative controls consisted of untreated cells. As a positive apoptotic control, cells were incubated with 10 μM campthotecin. The percentage of apoptosis was defined as the percent of YO-PRO-1 stained cells within the cell population of interest. Data were recorded for a total of 10,000 events per sample and analyzed.

### Intracellular calcium concentration assay

After subjection to different treatment regimens with DOX or DOX-TRF in 96-well plates, the respective cells were incubated with 7 μM fluo-4 AM as reported before [18], previously dissolved in DMSO for 60 min at 37 °C. The resulting fluorescence intensity was analyzed using a Fluoroskan Ascent FL microplate reader (Labsystems, Sweden) at 485 nm excitation and 525 nm emission wavelengths.

### Mitochondrial membrane potential assay

Changes in mitochondrial membrane potential were assessed by measuring the 1,1′,3,3′-tetraethylbenzimidazolcarbocyanine iodide (JC-1) fluorescence intensity ratio as reported before [19]. To this end, the cells were pre-incubated with 5 μM JC-1 in HBSS (140 mM NaCl, 5 mM KCl, 0.8 mM MgCl_2_, 1.8 mM CaCl_2_, 1 mM Na_2_HPO_4_, 10 mM HEPES and 1 % glucose) for 30 min. Next, the cells were centrifuged (300 *g* for 30 min at 22 °C) and washed twice with HBSS. The resulting fluorescence was measured on a Fluoroskan Ascent plate reader with filter pairs of 530 nm/590 nm and 485 nm/538 nm. The results are presented as the fluorescence ratio measured at 530 nm/590 nm to that measured at 485 nm/538 nm (dimer to monomer fluorescence) relative to the control untreated cell fluorescence ratio, which was set at 100 %.

### Cytochrome *c* release assay

To determine mitochondrial cytochrome *c* release, the cytosolic fractions of the cells were first separated from the mitochondrial fractions according to the manufacturer’s procedures (BioVision, USA). Next, the cells were exposed to DOX-TRF or DOX for 6, 12 or 24 h and collected by centrifugation at 600 *g* for 5 min at 4 °C. After homogenizing the cell pellets with cytosol extraction buffer (BioVision, USA) on ice for 40 min, the supernatant (cytosolic protein fraction) was immediately stored at −70 °C until use for biochemical analysis.

The level of cytochrome *c* in the cytosolic fraction was measured using a Cytochrome *c* Assay kit (R&D Systems, USA). This colorimetric assay was performed in clear 96-well plates pre-coated with an anti-cytochrome *c* monoclonal antibody, according to the manufacturer’s instructions [20]. Finally, the plates were read at 450 nm using a Cary 50 microplate reader (Cary, Melbourne, Australia).

### Statistical analyses

The data are expressed as a means ± S.D. Analysis of variance (ANOVA) with Tukey post hoc test was used for multiple comparisons. All statistical parameters were calculated using the STATISTICA program (StatSoft, Tulsa, OK, USA). A *p* value of < 0.05 was considered significant. The viability curves were fitted according to the sigmoidal dose–response model (four-parameter logistic equation) using the GraphPad Prism 4.03 software (GraphPad Inc).

## Results

### Cytotoxicity of DOX and DOX-TRF in normal and leukemia-derived cells

**Fig. 1:**
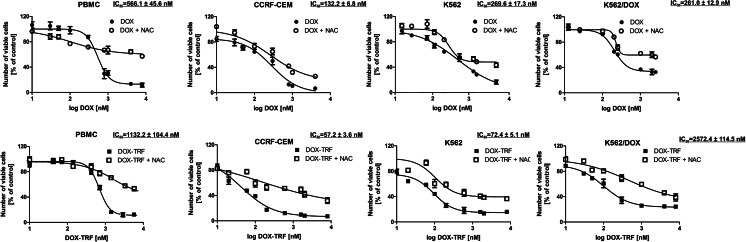
Representative dose–response curves following exposure to DOX versus DOX-TRF of PBMC, CCRF-CEM, K562 and K562/DOX cells +/− NAC. Cell survival was assessed by XTT assay. (*) *p* < 0.05, the effect of DOX and DOX-TRF on the viability of leukemia-derived and PBMC cells; (#) *p* < 0.05 changes between the samples pre-incubated with NAC. The values represent the mean ± SD of five independent experiments

The cytotoxicity of DOX and DOX-TRF was assessed in normal peripheral blood-derived mononuclear cells (PBMCs) and the leukemia-derived cell lines CCRF-CEM, K562 and K562/DOX using a XTT assay. We found that both compounds caused a dose-dependent decrease in cell viability in the normal and leukemia-derived cells (Fig. 1). DOX-TRF was two times less cytotoxic to the normal PBMC than DOX. In contrast, the IC_50_ of DOX-TRF was significantly lower than that of DOX in the leukemia-derived cells. The mean IC_50_ ratios were 2.4 for CCRF-CEM and 3.7 for K562. The cytotoxic effect of DOX-TRF over DOX was even more marked in the K562/DOX cells (IC_50_ ratio of 9.9). Pre-incubation of the leukemia-derived cells with the antioxidant NAC at 3 mM resulted in a partial protection from DOX-TRF cytotoxicity (~12–30 %), but more than that from DOX cytotoxicity (~5–15 %).

### ROS production changes in DOX and DOX-TRF treated normal and leukemia-derived cells

The time-course of reactive oxygen species (ROS) production was assessed after incubation of normal PBMCs and the leukemia-derived cell lines CCRF-CEM, K562 and K562/DOX with DOX or DOX-TRF. A significant increase of DCF fluorescence (see materials and methods) in PBMC was observed after a 3–24 h incubation with DOX, and 24 h after incubation with DOX-TRF (Table 1). ROS generation in the leukemia-derived cells was maximal after a 12 h treatment with DOX-TRF and increased ~1.3-fold in CCRF-CEM cells and ~1.5-fold in K562 cells. No significant differences in ROS levels were observed between the DOX and DOX-TRF-treated human leukemia-derived cells. However, DOX-TRF induced a 1.3-fold higher ROS production in K562/DOX cells than DOX. In order to confirm that the ROS production was related to the cytotoxicity of DOX and DOX-TRF, the cells were treated with the antioxidant NAC. We found that pretreatment of the cells with 3 μM NAC efficiently reduced the drug-induced increases in ROS levels.

**Table 1:** Comparison of the effects of DOX and DOX-TRF on ROS production in PBMC and leukemia-derived cells

Time of incubation [h]	DOX	DOX + NAC	DOX-TRF	DOX-TRF + NAC
CCRF-CEM				
3	108.72 ± 8.41	103.17 ± 11.07	100.89 ± 5.50	97.82 ± 4.82
6	121.90 ± 4.65*	92.50 ± 8.22^+^	126.27 ± 4.95*	93.99 ± 12.60^+^
12	134.91 ± 5.78*	108.79 ± 8.63^+^	129.09 ± 2.86*	103.62 ± 4.45^+^
24	107.37 ± 3.49	99.85 ± 2.60	108.20 ± 4.90	102.00 ± 5.54
48	105.55 ± 3.88	95.33 ± 7.43	98.75 ± 10.17	96.11 ± 7.41
72	98.25 ± 5.64	101.23 ± 7.25	112.37 ± 4.72	95.82 ± 10.83
PBMC				
3	122.58 ± 11.55*	114.29 ± 10.90	105.93 ± 11.95	103.66 ± 12.88
6	126.24 ± 10.53*	103.06 ± 6.37	107.17 ± 4.54	103.5 ± 2.68
12	118.23 ± 0.94*	94.07 ± 2.06	104.79 ± 1.57	91.83 ± 2.012
24	135.14 ± 9.82*	109.8 ± 1.92^+^	125.94 ± 6.84*	107.4 ± 6.74^+^
48	106.47 ± 3.12	93.67 ± 4.57	96.65 ± 4.27	95.31 ± 4.54
72	104.94 ± 5.095	107.23 ± 7.73	107.05 ± 2.039	108.15 ± 9
K562				
3	118.67 ± 5.69*	101.76 ± 3.64^+^	94.61 ± 1.04	90.44 ± 1.25
6	130.65 ± 3.50*	106.24 ± 2.62^+^	119.02 ± 2.33*	103.00 ± 1.65^+^
12	142.34 ± 4.53*	96.94 ± 2.04^+^	152.27 ± 7.49*	89.50 ± 11.88^+^
24	101.43 ± 4.17	86.82 ± 3.72	132.81 ± 1.88*	103.59 ± 3.67^+^
48	93.17 ± 7.04	97.12 ± 6.36	105.42 ± 6.68	104.53 ± 10.8
72	101.62 ± 2.55	99.15 ± 3.34	105.85 ± 4.04	102.49 ± 7.66
K562/DOX				
3	98.89 ± 2.86	99.85 ± 3.27	96.05 ± 2.9	98.92 ± 1.92
6	118.10 ± 3.45*	97.65 ± 3.28^+^	124.51 ± 4.16*	97.35 ± 4.41^+^
12	113.90 ± 13.61*	91.06 ± 8.57^+^	143.30 ± 6.74*^#^	87.01 ± 9.96^+^
24	98.22 ± 2.51	96.14 ± 5.41	117.67 ± 12.69*^#^	99.96 ± 1.61^+^
48	106.74 ± 2.69	101.93 ± 7.29	101.63 ± 6.09	82.41 ± 8.57
72	102.48 ± 8.5	82.87 ± 6.84	105.09 ± 11.24	87.98 ± 7.39

Cells were treated with IC_50_ doses of DOX or DOX-TRF for 3, 6, 12, 24, 48 and 72 h. The intensity of DCF fluorescence in the control cells was set at 100 % (data not shown). Each value represents the average ± SD of four independent experiments

*significantly different compared to control cells, *p* < 0.05

^+^significant changes compared to samples pre-incubated with NAC and subsequently incubated with drugs, *p* < 0.05

^#^significant differences between samples incubated with DOX and samples incubated with DOX-TRF, *p* < 0.05

### Apoptosis and necrosis changes in DOX and DOX-TRF treated normal and leukemia-derived cells

**Fig. 2:**
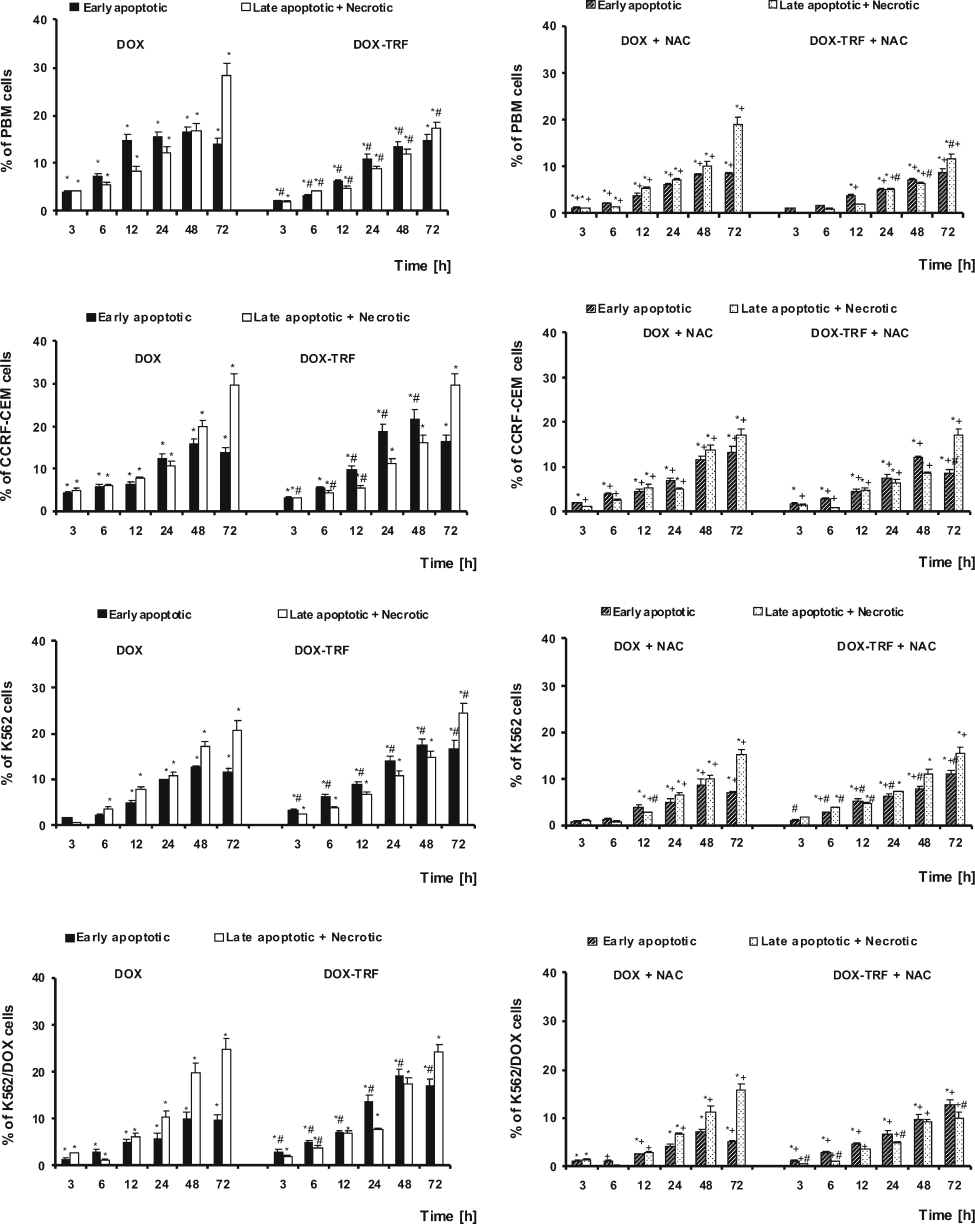
The effect of DOX and DOX-TRF without (*left panel*) and with (*right panel*) NAC on the level of early and late apoptotic and necrotic cells. NAC was added 1 h before DOX or DOX-TRF treatment. The number of cells in apoptosis and necrosis were counted by flow cytometry using YO-PRO-1 iodide/PI double staining, after which the percentage fractions of the respective cells were calculated. Data are mean ± SD of five samples. * *p* < 0.05 indicates significant differences between drug-treated and control (untreated) cells; # *p* < 0.05 indicates significant differences between cells treated with DOX and DOX-TRF; + *p* < 0.05 indicates significant differences between DOX or DOX-TRF treated cells and samples pre-incubated with NAC

**Fig. 3:**
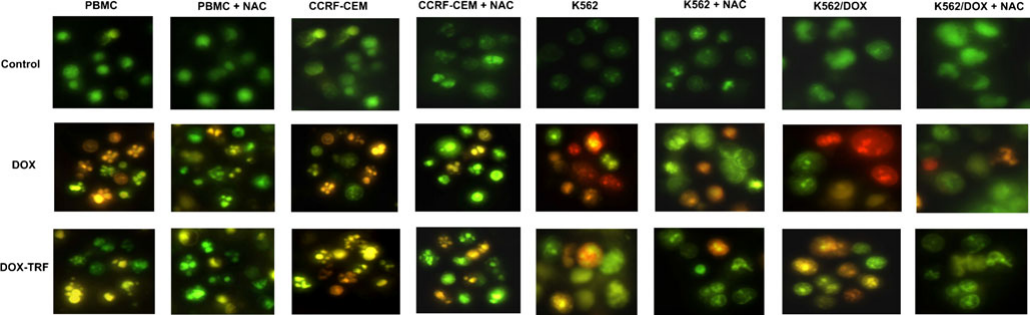
Morphological changes of PBMC, CCRF-CEM, K562 and K562/DOX cells at 48 h after DOX or DOX-TRF treatment +/− NAC. The cells were stained with PI and YO-PRO. In case of NAC treatment, cells were pre-incubated for 1 h, then DOX or DOX-TRF was added and the incubation was continued for up to 48 h. The cells were analyzed under a fluorescence microscope at 400× magnification

The ability of DOX and DOX-TRF to induce apoptosis or necrosis in normal PBMCs and the leukemia-derived cell lines CCRF-CEM, K562 and K562/DOX was estimated after treatment of the cells up to 72 h with doses corresponding to the respective IC_50_ values. Subsequent flow cytometry after YO-PRO-1/PI double staining allowed the distinction of three cell populations: living cells (green fluorescence), apoptotic cells (yellow fluorescence) and necrotic cells (red-brown fluorescence) (Fig. 2). In parallel, cellular morphologies were assessed using fluorescence microscopy. By doing so, we found that DOX-TRF more efficiently induced apoptotic hallmarks, such as chromatin condensation and nuclear fragmentation, than DOX (Fig. 3).

After shorter exposure times (3 h and 6 h), the levels of apoptosis and necrosis in both the normal and the leukemia-derived cells did not exceed 4–5 % of the total cell numbers. Incubation of the cells for 12–72 h with DOX or DOX-TRF increased the levels of apoptotic and necrotic cells. As shown in Fig. 2, treatment of PBMC with DOX or DOX-TRF caused an increase in the number of necrotic cells of up to 30 % after 72 h treatment with the free drug (DOX). The leukemia-derived cell lines exposed to DOX-TRF reached maximal values in the number of apoptotic cells (21.6 % for CCRF-CEM and 17.5 % for K562) after a 48 h incubation period. In K562/DOX cells the percentage of apoptosis was highest at 48 h with both drugs (19.2 % for DOX-TRF and 10.1 % for DOX), confirming that these DOX-resistant cells treated with the conjugate died mostly from apoptosis. As shown in Fig. 3, we found that the number of apoptotic and necrotic cells in the normal and leukemia-derived cells decreased in the presence of NAC. These latter results suggest that ROS is involved in the drug-induced apoptosis.

### Intracellular Ca^2+^ concentration changes in DOX and DOX-TRF treated normal and leukemia-derived cells

To investigate whether intracellular calcium ion changes were involved in the apoptosis induced by DOX-TRF or DOX, we assessed the level of free Ca^2+^ using the fluorescence probe Fluo-4-AM. By doing so, we found that in PBMC after a 24 h incubation with DOX and DOX-TRF the largest increases in free cytoplasmic Ca^2+^ were similar, reaching a level of ~26 % (Table 2). In the leukemia-derived cells, DOX-TRF induced an increase in intracellular free Ca^2+^ during the first 24 h. At this time point, the level of Ca^2+^ in the respective leukemia-derived cells treated with IC_50_ DOX-TRF increased by approximately 30, 33 and 25 %, respectively (*p* < *0.05*; Table 2). Administration of the free radical scavenger NAC resulted in a marked reduction in the DOX-TRF and DOX-induced changes in intracellular Ca^2+^.

**Table 2:** Effect of DOX and DOX-TRF on intracellular Ca^2+^ levels in PBMC and leukemia-derived cells

Time of incubation[h]	DOX	DOX + NAC	DOX-TRF	DOX-TRF + NAC
CCRF-CEM				
3	95.67 ± 2.86	96.95 ± 7.52	95.67 ± 2.86	90.45 ± 3.39
6	109.09 ± 12.86	94.50 ± 8.22	121.90 ± 4.65	93.99 ± 7.60
12	125.10 ± 6.75*	102.74 ± 2.84^+^	123.61 ± 8.86*	92.37 ± 7.78^+^
24	111.47 ± 3.62	98.23 ± 8.64	125.61 ± 4.90*	99.34 ± 6.87^+^
48	94.00 ± 6.70	103.00 ± 13.48	102.00 ± 13.40	101.00 ± 7.91
72	98.58 ± 1.50	100.40 ± 2.70	98.57 ± 5.64	100.8 ± 9.49
PBMC				
3	99.35 ± 3.02	100.85 ± 12.35	100.06 ± 10.00	102.65 ± 7.69
6	111.17 ± 7.21	91.55 ± 14.51	94.29 ± 14.53	97.55 ± 5.85
12	119.69 ± 8.67*	100.37 ± 4.52^+^	113.76 ± 0.81	100.35 ± 3.60
24	125.94 ± 10.41*	99.83 ± 10.61^+^	126.07 ± 4.13*	95.35 ± 4.47^+^
48	100.01 ± 3.50	102.71 ± 4.90	116.37 ± 1.58*	100.37 ± 1.49^+^
72	97.82 ± 4.08	103.75 ± 8.35	100.36 ± 1.52	87.9 ± 8.02
K562				
3	99.67 ± 2.86	96.95 ± 17.52	98.31 ± 12.37	95.45 ± 13.39
6	104.71 ± 6.88	101.01 ± 3.38	98.47 ± 9.89	97.45 ± 2.80
12	129.09 ± 9.86*	82.50 ± 8.22^+^	117.90 ± 4.65*	93.99 ± 12.60^+^
24	98.34 ± 6.49	103.80 ± 5.39	133.43 ± 7.36*^#^	105.47 ± 1.83^+^
48	101.16 ± 5.41	97.92 ± 3.40	98.81 ± 3.00	102.08 ± 7.49
72	99.27 ± 5.73	94.63 ± 7.62	110.90 ± 4.35	102.34 ± 4.16
K562/DOX				
3	99.5 ± 7.90	102.90 ± 3.40	98.10 ± 4.70	100.60 ± 6.80
6	119.9 ± 6.70	101.80 ± 5.10	95.30 ± 8.10	97.30 ± 4.90
12	122.62 ± 1.42	97.53 ± 1.76	126.81 ± 9.53*	96.54 ± 3.84^+^
24	109.46 ± 9.04	99.28 ± 5.60	133.42 ± 11.40*^#^	90.05 ± 8.40^+^
48	92.42 ± 11.40	97.85 ± 6.20	118.11 ± 10.76*	97.04 ± 5.83^+^
72	97.65 ± 10.48	97.05 ± 6.27	107.44 ± 13.05	98.81 ± 7.47

The intensity of Fluo- 4 AM fluorescence in the control cells was set at 100 % (data not shown). Each value represents the mean ± SD of three independent experiments

*significantly different compared to control cells, *p* < 0.05

^+^significant differences between DOX or DOX-TRF treated cells and samples pre-incubated with NAC, *p* < 0.05

^#^significant differences between samples incubated with DOX and samples incubated with DOX-TRF, *p* < 0.05

### Mitochondrial membrane potential changes in DOX and DOX-TRF treated normal and leukemia-derived cells

Mitochondrial alterations after DOX or DOX-TRF treatment were assessed by measuring changes in mitochondrial membrane potential (Δψ_m_) in normal PBMCs and the leukemia-derived cell lines CCRF-CEM, K562 and K562/DOX using the fluorescence probe JC-1. As a positive control, cells were pre-incubated with CCCP, a protonophoric uncoupler of oxidative phosphorylation, prior to JC-1 labeling, [21]. Upon incubation with CCCP, a profound fall in membrane potential was observed as expected, which was reflected by a decrease in the JC-1 dimer to JC-1 monomer fluorescence ratio (data not shown).

**Fig. 4:**
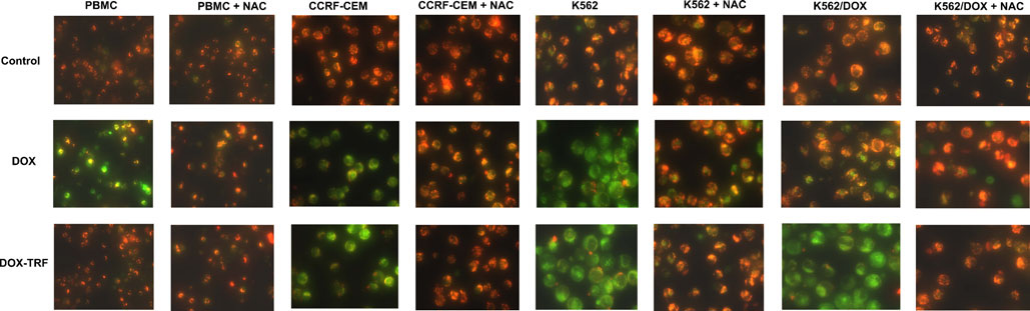
Fluorescent microscopy images of control cells, incubated with PBS, and cells treated with IC_50_ concentrations of DOX or DOX-TRF for 12 h at 37 °C +/− NAC. Red fluorescence of JC-1 dimers is present in cell areas with a high mitochondrial membrane potential, while green fluorescence of JC-monomers is prevalent in cell areas with low a mitochondrial membrane potential. The cells were analyzed under a fluorescence microscope at 400× magnification

We found that DOX and DOX-TRF induced time-dependent changes in Δψ_m_ (Table 3). In normal cells, Δψ_m_ collapsed after a 6 h incubation with DOX (78 %) and after a 24 h incubation with DOX-TRF (86 %). The fluorescence intensity of JC-1 decreased by 21 % at 6 h in CCRF-CEM cells and by 26 % at 12 h in K562 cells, whereas in k562/DOX cells a maximal drop of Δψ_m_ was detected after a 24 h incubation with DOX-TRF. After prolonged incubations (48–72 h), the fluorescence intensity of JC-1 in all cell lines increased to nearly 95 % compared to the control value. Pre-incubation of the cells with NAC revealed a protective effect, which indicates that the drop in mitochondrial membrane potential was ROS-dependent. Additionally, we confirmed the Δψ_m_ changes in drug-treated cells using fluorescence microscopy. Figure 4 shows that DOX-TRF treatment caused a remarkable increase in green fluorescence of JC-1 monomers in the leukemia-derived cells, indicating a reduction in mitochondrial membrane potential. In contrast, the red fluorescence of JC-1 dimers was seen mainly in untreated (control) cells with a high mitochondrial potential.

**Table 3:** Mitochondrial membrane potential changes in PBMC and leukemia-derived cells after treatment with DOX or DOX-TRF

Time of incubation [h]	DOX	DOX + NAC	DOX-TRF	DOX-TRF + NAC
CCRF-CEM				
3	98.90 ± 9.00	98.84 ± 2.70	97.81 ± 1.94	99.80 ± 10.50
6	79.95 ± 8.94*	97.16 ± 3.13^+^	82.84 ± 4.58*	98.63 ± 5.02^+^
12	75.10 ± 6.75*	102.74 ± 2.84^+^	72.61 ± 2.86*	101.34 ± 7.78^+^
24	98.47 ± 3.62	98.23 ± 8.64	95.61 ± 4.90	99.34 ± 6.87
48	94.00 ± 6.70	103. 00 ± 13.48	102. 00 ± 13.40	101. 00 ± 7.91
72	98.58 ± 1.50	100.40 ± 2.70	98.57 ± 5.64	100.80 ± 9.49
PBMC				
3	107.75 ± 5.63	105.34 ± 4.40	100.51 ± 10.77	106.93 ± 6.10
6	78.33 ± 8.78*	92.91 ± 2.24	107.98 ± 7.30	104.96 ± 9.04
12	80.58 ± 4.56*	102.53 ± 4.42^+^	97.90 ± 4.88	104.24 ± 5.58
24	89.34 ± 3.65	102.18 ± 3.28	86.61 ± 5.21*	104.53 ± 1.24^+^
48	100. 00 ± 4.68	106.04 ± 5.77	95.97 ± 5.19	102.65 ± 3.73
72	109.71 ± 4.27	104.30 ± 2.70	95.19 ± 3.19	109.00 ± 7.28
K562				
3	98.58 ± 1.50	100.40 ± 2.70	98.57 ± 5.64	100.80 ± 9.49
6	80.91 ± 5.11*	102.00 ± 3.40^+^	83.34 ± 4.60*	99.30 ± 5.60^+^
12	79.93 ± 5.89*	101.19 ± 9.09+	74.87 ± 4.68*	97.31 ± 5.36+
24	90.00 ± 6.22	100.98 ± 3.84	83.00 ± 4.41*	102.78 ± 5.42^+^
48	93.06 ± 7.81	98.92 ± 9.46	97.91 ± 3.37	97.36 ± 2.60
72	98.74 ± 7.39	101.92 ± 5.13	92.78 ± 5.90	101.18 ± 4.49
K562/DOX				
3	100.68 ± 2.52	106.39 ± 4.09	93.27 ± 3.94	103.21 ± 2.57
6	96.97 ± 0.74	100.78 ± 10.39	99.95 ± 14.09	99.89 ± 6.26
12	80.07 ± 4.17*	98.07 ± 3.03^+^	75.48 ± 5.16*	94.38 ± 6.64^+^
24	85.25 ± 4.93*	101.61 ± 7.44^+^	67.95 ± 5.53*^#^	103.37 ± 6.88^+^
48	108.60 ± 2.32	109.75 ± 1.17	93.89 ± 2.97	104.78 ± 5.50
72	104.36 ± 1.70	99.83 ± 6.99	102.44 ± 7.36	100.20 ± 3.90

Each value represents the mean ± SD of six separate experiments

*significantly different compared to the respective control cells (taken as 100 %, data not shown), *p* < 0.05

^+^significant differences between DOX or DOX-TRF treated cells and samples pre-incubated with NAC, *p* < 0.05

^#^significant differences between samples incubated with DOX and samples incubated with DOX-TRF, *p* < 0.05

### Release of cytochrome c from mitochondria in DOX and DOX-TRF treated normal and leukemia-derived cells

**Fig. 5:**
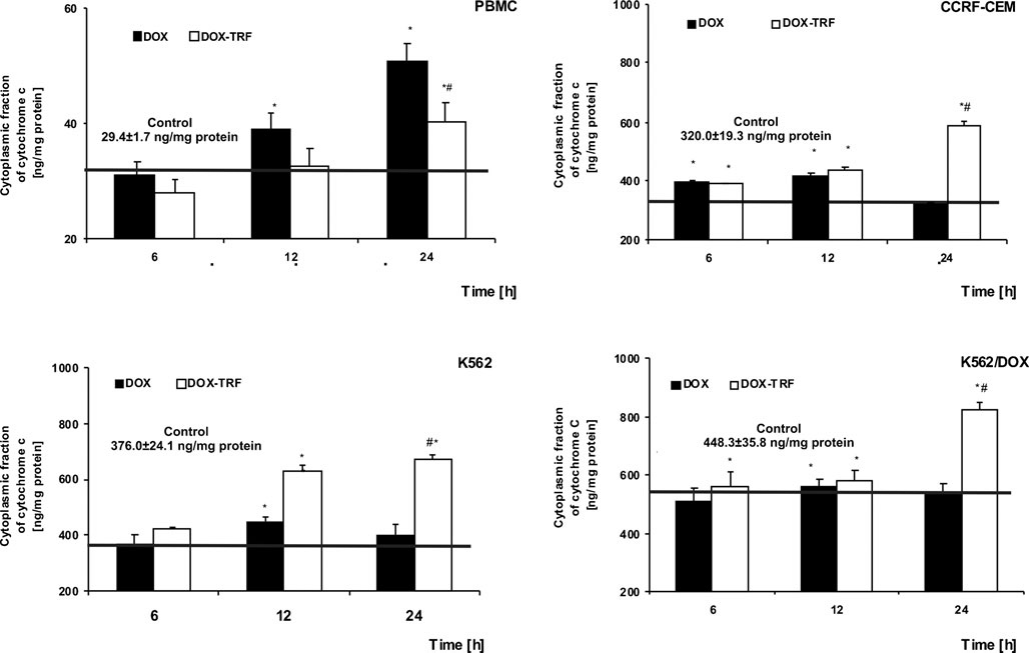
Release of cytochrome *c* from mitochondria in PBMC and leukemia-derived cells after treatment with DOX or DOX-TRF. Results represent the mean ± SD of three independent experiments. * *p* < 0.05 indicates significantly different from the control; # *p* < 0.05 indicates significant difference between the samples incubated with DOX and DOX-TRF

We monitored the efflux of cytochrome *c* from mitochondria to cytosol using an ELISA assay kit at 6, 12 and 24 h in DOX-TRF or DOX-treated normal PBMCs and the leukemia-derived cell lines CCRF-CEM, K562 and K562/DOX. We found that DOX induced a gradual increase in cytochrome *c* in normal cells after a 12 h (38.9 ng/mg protein) and a 24 h (50.8 ng/mg protein) incubation period (Fig. 5). DOX-TRF treatment of PBMC led to an increase in cytochrome *c* (40.2 ng/mg protein) after a 24 h incubation period. As shown in Fig. 5, DOX-TRF induced the highest increases in cytosolic cytochrome *c* after 24 h in CCRF-CEM and K562 cells (590.1 and 679.0 ng/mg protein), whereas a maximal level of cytochrome *c* was released from the mitochondria after a 12 h incubation with DOX (415.5 and 451.6 ng/mg protein in CCRF-CEM and K562 cells, respectively).

In K562/DOX cells, DOX-TRF caused a release of cytochrome *c* from mitochondria at all time points tested, while DOX led to a maximal increase in cytosolic cytochrome *c* at 12 h, reaching 560.5 ng/mg protein. It appears, therefore, that the cytosolic level of cytochrome *c* did not correlate with the sensitivity of the cells to these compounds. Our studies do confirm a time-dependent relationship between the decrease in mitochondrial membrane potential and the release of cytochrome *c* in cells treated with DOX-TRF.

## Discussion

A major reason to find a way to deliver anticancer drugs directly to neoplastic cells is the rapid emergence of drug resistant cell populations [14]. Ample literature data indicate that over-expression of ATP-binding cassette (ABC) proteins is closely related to increases in drug resistance [22–24]. Clearly, there is a strong need to develop new ways to overcome drug resistance e.g. by using specific drug carriers, such as liposomes, dendrimers, nanoparticles or plasma proteins [25].

Previously, we showed that there is a relationship between an increased cytotoxicity of the DOX-TRF conjugate and its effect on the functionality of P-glycoprotein, one of the most important ABC transporters [14]. We anticipate that due to a different mode of action of the DOX-TRF conjugate, it may be able to circumvent therapeutic complications such as cardiotoxicity, nephrotoxicity and drug resistance. As yet, however, little is known about the type of cell death provoked by DOX-TRF. Therefore, we set out to analyze the induction of cell death by DOX-TRF in relation to the generation of free radicals (ROS). We found that DOX-TRF was able to induce both apoptosis and necrosis in the cells tested. Prolonged incubation with DOX-TRF resulted in a higher apoptotic rate compared to a similar incubation with DOX. ROS generation was found to be partially associated with DOX-TRF and DOX cytotoxicity. In DOX-TRF or DOX treated leukemia-derived cells, ROS were produced partly as normal metabolic products and partly through the activation of ROS-producing enzymes. DOX-TRF was most toxic in K562/DOX cells, which are resistant to DOX. K562 and CCRF-CEM cells were 2.6 and 4 times less sensitive to DOX than to DOX-TRF. In K562/DOX cells, DOX-TRF was 10-fold more cytotoxic than DOX. Its IC_50_ in normal blood cells exceeded 1000 nM, indicating that DOX-TRF may cause greater harm to leukemic cells than to PBMC.

It has amply been shown that ROS generation by anticancer drugs and the resulting oxidative stress are involved in the initiation and/or execution phases of apoptosis [26, 27]. Here we show that addition of the free radical scavenger NAC to the DOX formulations significantly decreased the fraction of apoptotic and necrotic cells, including the apoptotic features. Our results are in agreement with those of Rogalska et al. [18], who showed that aclarubicin was able to generate free radicals and, as a consequence, to affect the apoptotic process in human solid tumors. Given that anthracyclines encompass both para-quinone and para-hydroquinone moieties, ROS generation may be initiated through reduction or oxidation of the drug [21, 28].

ROS levels were assessed through 2′,7′-dichlorodihydrofluorescein diacetate (DCF) fluorescence measurements. We found that DOX-TRF treatment led to the production of ROS after a prolonged incubation time in leukemic cells, while DOX caused an increase of DCF fluorescence after 6 and 12 h incubation in the leukemia-derived cell lines and after a 24 h incubation in PBMCs. A lower toxicity of free radicals to normal cells (i.e., cardiomyocytes and endothelial cells) has previously been observed after incubation with an epirubicin conjugate of polyethyleneglycol (PEG) and nitric oxide. While this compound was toxic to Caco2 cancer cells, normal cells remained intact during treatment [29].

Part of our current study was focused on the biochemical changes induced during apoptosis by DOX-TRF in normal and leukemia-derived cells. We investigated mitochondrial membrane potential, the level of intracellular Ca^2+^ and the intracellular fraction of cytochrome *c*. Dissipation of Δψ_m_ is one of the markers for a mitochondrial involvement in apoptosis [30]. Our data show that a drop in mitochondrial membrane potential (Δψ_m_) occurred during the first 6 h following DOX or DOX-TRF treatment in the leukemia-derived cell lines tested. In PBMC and in the doxorubicin-resistant erythroleukemia cell line K562/DOX, the Δψ_m_ decreased after 24 and 12 h of DOX-TRF treatment, respectively. After a prolonged incubation time (48–72 h), irrespective of cell type, the Δψ_m_ gradually recovered. These observations suggest that Δψ_m_ loss is an early event in the apoptotic cascade, induced by both DOX and DOX-TRF. We also found that DOX-TRF was more effective than DOX in the depolarization of mitochondria in malignant cells. Previously, a decrease in Δψ_m_ was also observed after DOX-induced apoptosis in human mammary adenocarcinoma cells (MTLn3), in porcine renal proximal tubular cells (LLC-PK1) [31] and in various other human tumor cells after amrubicin treatment [32].

It has amply been suggested that free Ca^2+^ is one of the most important signaling agents in human cancer cells [33]. A rise in intracellular Ca^2+^ may be one of the triggering events that lead to cell damage or apoptosis [34]. Kania et al. [35] have shown that aclarubicin can induce an increase in the intracellular pool of Ca^2+^ after a 4 h incubation of normal (S2) and trisomic (BB) diabetic fibroblasts, whereas Brachwitz et al. [36] observed an increase in Ca^2+^ in the human mammary tumor cell line H184 treated with ara-cytidine-59-alkylphosphono-phosphates. Here, we found that the cellular Ca^2+^ levels rapidly increased upon DOX and DOX-TRF treatment, which was accompanied by Δψ_m_ reduction in all cell types tested.

Another crucial event in the path to apoptosis is the release of cytochrome *c* from mitochondria [36]. Cytochrome *c* is normally located in the intermembranous space of the mitochondria, loosely bound to the inner membrane. Several authors have reported a release of cytochrome *c* to the cytosol upon DOX treatment [37]. Although apoptosis can occur via cytochrome *c*-independent mechanisms, it is well established that in most cell types, once cytochrome *c* is released into the cytosol, it interacts with APAF1 and procaspase-9, leading to the generation of active caspase-9, which is capable to proteolytically activate caspase-3 [38, 39]. As shown by many authors, anthracyclines can induce apoptosis in cancer cells by increasing the cytosolic fraction of cytochrome *c* [40]. Here, we found that both DOX-TRF and DOX can cause an efflux of cytochrome *c* from mitochondria.

The ability of DOX to release cytochrome *c* into the cytosol has been documented by Childs et al. [41]. They reported that the assessment of apoptosis by determination of the amount of cytosolic cytochrome *c* indicated that doxorubicin can induce oxidative stress and mitochondria-mediated apoptosis, as well as adaptive responses by mitochondria to protect cardiac myocytes in vivo.

We conclude that DOX-TRF can induce ROS-dependent changes characteristic of apoptosis in human leukemia-derived cells. We found that, compared to DOX, DOX-TRF was less toxic to normal peripheral blood mononuclear cells and significantly more cytotoxic to leukemia-derived cells. Transferrin (TRF) serves a promising and efficacious carrier for the targeted delivery of doxorubicin.
